# A Sulfated Polysaccharide from the Green Alga *Caulerpa taxifolia*: Characteristics of Its Structure and Anti-Diabetic Activity

**DOI:** 10.3390/md23100374

**Published:** 2025-09-25

**Authors:** Shan Liu, Ling Qin, Dan Li, Fang Lu, Mengdi Liang, Jiejie Hao

**Affiliations:** 1 Key Laboratory of Marine Drugs of Ministry of Education, School of Medicine and Pharmacy, Ocean University of China, Qingdao 266003, China; 2 Marine Science Research Institute of Shandong Province, Qingdao 266104, China

**Keywords:** sulfated galactan, *Caulerpa taxifolia*, structural characterization, anti-diabetic activity

## Abstract

Polysaccharides derived from green algae have garnered significant attention owing to their distinctive structural characteristics and biological activities. In particular, sulfated polysaccharides from these algae represent a promising frontier in the discovery of novel therapeutic agents. In this present study, a sulfated galactan from *Caulerpa taxifolia*, designated SGC, was obtained by dilute alkali extraction and chromatographic purification. On the basis of chemical and spectroscopic analyses, the backbone of SGC was constituted by a backbone of →3)-β-d-Gal*p*-(1→ with sulfate substitution at the C-2 and a branch on C-6. The side chains contained →6)-β-d-Gal*p*(2SO_4_)-(1→, →6)-β-d-Gal*p*(3OMe)-(1→ and →3)-β-d-Gal*p*(4,6-Pyr)-(1→ units. SGC possessed strong hypoglycemic activity in vitro, as evaluated by an assay of α-amylase inhibition. The anti-diabetic activity of SGC in vivo was further investigated using T2DM mice induced by high-fat diet combined with streptozotocin. The results indicated that SGC markedly restored body weight, reduced fasting blood glucose and possessed a significant glucose-regulating effect. Furthermore, SGC effectively increased insulin sensitivity and mitigated insulin resistance. Additionally, SGC effectively regulated lipid metabolism and alleviated oxidative stress. Notably, SGC ameliorated liver and pancreas damage induced by high-fat diet combined with streptozotocin. The investigation demonstrates that SGC is a unique sulfated galactan and has potential as a novel anti-diabetic agent.

## 1. Introduction

The mounting global burden of type 2 diabetes mellitus (T2DM) is exemplified by significant epidemiological transitions in China, characterized by both increasing prevalence and progressively earlier age of disease onset. T2DM has emerged as a critical global public health challenge, necessitating the urgent formulation and implementation of targeted interventions [1,2,3]. While dietary regulation and structured exercise regimens constitute the foundational framework of contemporary diabetes management, pharmacological research has progressively emphasized the development of molecularly precise therapeutic agents to optimize treatment efficacy [4,5]. Current clinical practice utilizes conventional therapeutic agents such as metformin, sulfonylureas, α-glucosidase inhibitors and SGLT2 inhibitors as principal therapeutic modalities for glycemic regulation. However, their therapeutic application remains limited by adverse side effect profiles, notably gastrointestinal complications and risks of iatrogenic hypoglycemic episodes [6,7]. To address rising demand for anti-diabetic drugs, probing natural sources for active compounds is essential.

Marine-derived polysaccharides, particularly algal polysaccharides, represent a pharmacologically important category of bioactive compounds. Extensive investigations have confirmed their diverse biological functionalities, including anticoagulant activity, antineoplastic effects, antiviral action, scavenging of reactive oxygen species, modulation of glucose homeostasis and immunoregulatory properties [8]. It is worth mentioning that the extraction method affects the yield and activity of algal polysaccharides. Comparative evaluation of dynamic maceration and ultrasonic-assisted extraction of fucoidan from four arctic brown algae on its antioxidant and anticancer properties was recently reported [9]. In the past few years, the anti-diabetic effect of algal polysaccharides has attracted considerable attention. The polysaccharide SFP-2 from *Sargassum fusiforme*, which consists of fucose, mannose, rhamnose, glucose, galactose and glucuronic acid, markedly ameliorated hyperglycemia, hyperlipidemia and diabetes-associated hepatic and renal impairments [10]. The sulfated galactoarabinan OHSS2 from *Cladophora oligoclada*, which was constituted by a backbone of (1→4)-β-l-arabinopyranose units with partial sulfation at C-3, effectively protected mitochondrial function from human islet amyloid polypeptide aggregation-induced damage and had potential prospects as a novel anti-diabetic agent [11]. Sulfated galactan SPs from *Gracilaria gracilis* possessed inhibitory activity against key enzymes involved in carbohydrate and lipid digestion, promoted glucose uptake in yeast cells and psoas muscle tissue and attenuated glucose absorption in the intestinal epithelium [12]. The algal polysaccharides are one group of potential anti-diabetic agents.

The genus *Caulerpa* (*Caulerpaceae*, *Caulerpales*, *Chlorophyta*) has a cosmopolitan distribution in marine ecosystems. To date, limited research on the polysaccharides from the *Caulerpa* species can be found [13,14,15]. A spray-dried polysaccharide extracted from *Caulerpa lentillifera* exhibited significant antioxidant activity [16]. A polysaccharide extract from the seaweed *Caulerpa racemosa* (PCR) exhibits anti-diabetic and nephroprotective effects in high fructose-streptozotocin-induced diabetic nephropathy, which can be applied to prevent the progression of the disease [17]. In the present work, a sulfated galactan from the green alga *C. taxifolia* was extracted with dilute alkali and subsequently purified using anion-exchange and size-exclusion chromatography. The purpose of this study was to investigate the structural characteristics of the polysaccharide and evaluate its anti-diabetic activity.

## 2. Results and Discussion

### 2.1. Structural Characteristics of the Sulfated Polysaccharide SGC

**Figure 1:**
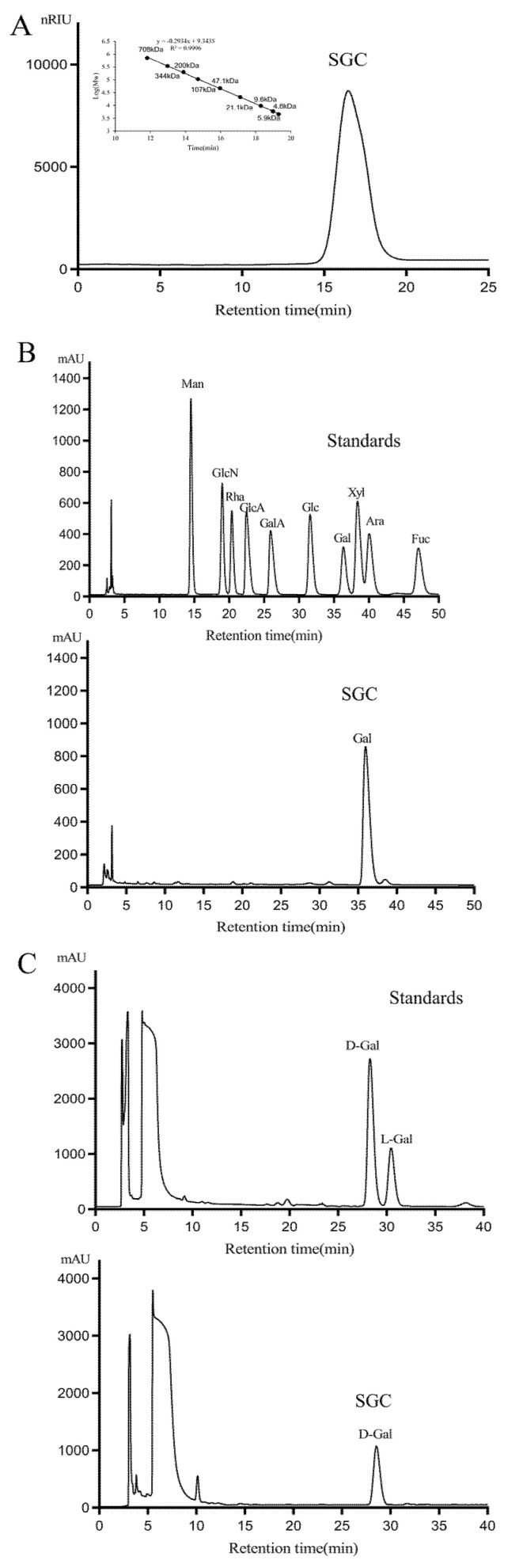
HPGPC and HPLC chromatograms of SGC. (**A**) HPGPC chromatogram of SGC on a ShodexOHpak SB-804 HQ column and the standard curve of molecular weight; (**B**) HPLC chromatogram for the monosaccharide composition analysis of SGC (Man: d-mannose, GlcN: d-glucosamine, Rha: l-rhamnose, GlcA: d-glucuronic acid, GalA: d-galacturonic acid, Glc: d-glucose, Gal: d-galactose, Xyl: d-xylose, Ara: l-arabinose, Fuc: l-fucose); and (**C**) HPLC chromatogram of the sugar configuration determination of SGC.

The polysaccharide SGC was obtained through dilute alkali extraction, ion-exchange and gel filtration chromatography purification. Alkali extraction is a well-established method for extracting polysaccharides from seaweed. The concentration of the alkaline solution, temperature and duration significantly affect the yield, composition and structure of the extracted polysaccharides [18,19,20]. Recently, a method combining squeezing followed by spray drying has been reported as a practical and scalable approach for producing polysaccharides [16]. SGC exhibited a monodisperse symmetrical peak in a high-performance gel permeation chromatography (HPGPC) chromatogram (Figure 1A), and its molecular weight was estimated to be 31.4 kDa. SGC contained 86.6% total sugar, 7.3% sulfate and 4.1% pyruvic acid, while uronic acid and protein were undetectable. High-performance liquid chromatography (HPLC) assay demonstrated that SGC consisted of galactose. (Figure 1B). Sugar configuration analysis by HPLC indicated that the galactose in SGC was d-configuration (Figure 1C).

Methylation analysis provides useful information on the linkage patterns of sugar units, substitution positions of sulfate and pyruvate residues as well as branched features. Here, desulfated, depyruvylated, and fully desulfated and depyruvylated products of SGC, which were designated as dsSGC, dpSGC and dsdpSGC, respectively, were prepared. The sulfate content of dpSGC was estimated to be 7.1%, and no sulfate existed in dsSGC. No pyruvic acid was detected in dpSGC and dsdpSGC. Comparative methylation analyses among SGC, dsSGC, dpSGC and dsdpSGC were performed. In comparison with the data of SGC, →2,6)-Gal*p*-(1→ and →2,3,6)-Gal*p*-(1→ units disappeared in dsSGC, while the amounts of →6)-Gal*p*-(1→ and →3,6)-Gal*p*-(1→ units increased (Table 1). Thus, sulfate substitutions occurred at the C-2 of the →6)-Gal*p*-(1→ unit and the C-2 of the →3,6)-Gal*p*-(1→ unit, representing approximately 14% of the total glycosyl residues in SGC. Notably, compared with the data of SGC, dpSGC exhibited complete removal of →3,4,6)-Gal*p*-(1→ linkage, accompanied by an increase in the →3)-Gal*p*-(1→ unit, thereby confirming pyruvate substitution at the C-4 and C-6 of the →3)-Gal*p*-(1→ unit. This modification corresponded to approximately 6% pyruvate ketal-modified residue in SGC. From the data of dsdpSGC, it was deduced that the backbone of SGC was mainly composed of →3)-Gal*p*-(1→ residue. Furthermore, the consistent detection of →3,6)-Gal*p*-(1→ linkage across dsSGC, dpSGC and dsdpSGC indicated the presence of branches at either the C-3 or C-6 positions, constituting approximately 12% of the side-chain substitutions in SGC. Further NMR spectroscopic analysis was performed to elucidate the detailed branching features.

To further elucidate the structure of SGC, the NMR spectra of SGC and dsSGC were analyzed. NMR spectra of SGC are presented in Figure S1. The ^1^H NMR spectrum of SGC exhibited seven anomeric signals at δ_H_ 4.93 (A), 4.87 (B), 4.73 (C), 4.70 (D), 4.57 (E), 4.53 (F) and 4.51 (G). Correspondingly, the ^13^C NMR spectrum of SGC revealed anomeric carbon resonances at δ_C_ 105.44 (G), 104.77 (F), 104.76 (D), 104.45 (E), 103.93 (A) 103.26 (C) and 101.89 (B), indicating that galactose residues were β-anomer [21]. In addition, a methyl signal (δ_H_ 1.51) and carbonyl group signal (δ_C_ 177.62) were found. The carbon signal observed at δ_C_ 102.35 in the ^13^C NMR spectrum, which exhibited no corresponding correlation in the ^1^H–^13^C HSQC experiment, was conclusively assigned to the C-2 position of the acetal-linked pyruvic acid residue. Comprehensive analysis of the heteronuclear correlation spectra revealed critical structural evidence. The cross signal at δ_H_/δ_C_ 1.51/26.68 in the ^1^H–^13^C HSQC spectrum, combined with the long-range correlations observed at δ_H_/δ_C_ 1.51/102.35 and 1.51/177.62 in the ^1^H–^13^C HMBC spectrum, confirmed that the pyruvate formed a six-membered cyclic ketals with *O*-4 and *O*-6 of galactose unit, corresponding to the R isomer [22,23]. The structural elucidation of SGC was achieved through integrated analysis of ^1^H NMR, ^13^C NMR, ^1^H–^1^H COSY and ^1^H–^13^C HSQC spectra.

In the ^1^H–^13^C HSQC spectrum of SGC, residue A exhibited a characteristic H-1/C-1 correlation at δ_H_/δ_C_ 4.93/103.93. Downfield chemical shifts in H-2/C-2 (δH/δC 4.36/79.71) and H-6/C-6 (δ_H_/δ_C_ 4.00/69.89) were observed, allowing structural assignment of A as →6)-β-d-Gal*p*(2SO_4_)-(1→ [21]. Residue B displayed an H-1/C-1 correlation at δ_H_/δ_C_ 4.87/101.89. Characteristic downfield shifts for H-2/C-2 (δ_H_/δ_C_ 4.36/79.71), H-3/C-3 (δ_H_/δ_C_ 3.95/83.51) and H-6/C-6 (δ_H_/δ_C_ 4.05/68.39) were identified, confirming the structure of B as →3,6)-β-d-Gal*p*(2SO_4_)-(1→ [24]. Residue C showed an H-1/C-1 correlation at δ_H_/δ_C_ 4.73/103.26. Characteristic downfield shifts were observed for H-3/C-3 (δ_H_/δ_C_ 3.47/81.72) and H-6/C-6 (δ_H_/δ_C_ 4.09/69.89). The presence of an *O*-methyl group was confirmed by δ_H_/δ_C_ 3.51/57.86, with an additional ^1^H–^13^C HMBC correlation at δ_H_/δ_C_ 3.51/81.72. This confirmed the correlation between the *O*-methyl proton signal and the C-3 position of the sugar residue, indicating substitution of the C-3 position by an *O*-methyl group. Consequently, C was assigned to →6)-β-d-Gal*p*(3OMe)-(1→ [25]. Residue D exhibited an H-1/C-1 correlation at δ_H_/δ_C_ 4.70/104.76. Characteristic downfield shifts were observed for H-3/C-3 (δ_H_/δ_C_ 3.93/83.42) and H-6/C-6 (δ_H_/δ_C_ 3.94/69.96). Consequently, D was assigned to the structural motif →3,6)-β-d-Gal*p*-(1→ [21]. Residue E had an H-1/C-1 correlation at δ_H_/δ_C_ 4.57/104.45. A distinct downfield shift was identified for H-3/C-3 (δ_H_/δ_C_ 3.90/84.09). Based on these spectral features, E was conclusively assigned to the structural motif →3)-β-d-Gal*p*-(1→ [24]. Residue F presented an H-1/C-1 correlation at δ_H_/δ_C_ 4.53/104.77. A diagnostic downfield shift was found for H-3/C-3 (δ_H_/δ_C_ 4.38/77.25), consistent with substitution at the C-3 position. The pyruvate substitution site was unambiguously confirmed through the long-range ^1^H–^13^C HMBC correlation between H-6 (F) and the R-Pyr C-2 (δ_H_/δ_C_ 3.99/102.35). Additionally, characteristic resonances corresponding to H-6/C-6 (δ_H_/δ_C_ 3.99/66.67) of the pyruvate moiety were well-resolved in the ^1^H–^13^C HSQC spectrum, further corroborating the substitution pattern, thus F was assigned to →3)-β-d-Gal*p*(4,6-Pyr)-(1→ [23]. Residue G displayed an H-1/C-1 correlation at δ_H_/δ_C_ 4.51/105.44, thus G was assigned to β-d-Gal*p*-(1→ [21]. NMR data of SGC are summarized in Table 2.

NMR spectra of dsSGC are shown in Figure S2. The ^1^H NMR spectrum of dsSGC revealed six anomeric proton resonances at δ_H_ 4.72 (C), 4.71 (D), 4.55 (E), 4.52 (F), 4.50 (G) and 4.46 (H), with corresponding ^13^C NMR signals at δ_C_ 105.96 (G), 105.70 (E), 105.36 (C), 105.03 (H),104.78 (D) and 104.33 (F) in the anomeric region. The presence of an *O*-methyl group was confirmed by δ_H_/δ_C_ 3.51/57.79 (^1^H–^13^C HSQC). In addition, pyruvic acid residue signals at δн/δ_C_ 1.50/26.51 (^1^H–^13^C HSQC), δн/δ_C_ 1.50/102.39 and 1.50/177.04 (^1^H–^13^C HMBC) were found, confirming the presence of a 4,6-*O*-pyruvate ketal group. Combined with the NMR analysis results of SGC, integrated analysis of dsSGC NMR data enabled structural assignment of glycosidic linkages. In the ^1^H–^13^C HSQC spectrum, the downfield signals of H-3/C-3 (δ_H_/δ_C_ 3.47/81.62) and H-6/C-6 (δ_H_/δ_C_ 3.92,4.09/70.24) in residue C, along with a ^1^H–^13^C HMBC correlation at δ_H_/δ_C_ 3.51/81.62, suggest an *O*-methyl substitution at C-3 of the galactopyranosyl unit. Consequently, residue C was identified as →6)-β-d-Gal*p*(3OMe)-(1→. The downfield shifts in H-3/C-3 (δ_H_/δ_C_ 3.84/83.68) and H-6/C-6 (δ_H_/δ_C_ 3.97,4.09/70.95) in residue D confirmed its structural assignment as →3,6)-β-d-Gal*p*-(1→. The downfield signals of H-3/C-3 (δ_H_/δ_C_ 3.89/83.53) in residue E indicated its identification as →3)-β-d-Gal*p*-(1→. The presence of the signals H-3/C-3 (δ_H_/δ_C_ 4.29/79.75), H-4/C-4 (δ_H_/δ_C_ 4.20/72.44), H-6/C-6 (δ_H_/δ_C_ 3.98, 4.08/66.69) and δ_H_/δ_C_ 3.98/102.39 (^1^H–^13^C HMBC) confirmed residue F as →3)-β-d-Gal*p*-(4,6-Pyr)-(1→. G was identified as β-d-Gal*p*-(1→. Similarly, the downfield shifts in H-6/C-6 (δ_H_/δ_C_ 3.92, 4.09/70.24) in residue H supported its assignment as →6)-β-d-Gal*p*-(1→ [21]. NMR data of dsSGC are summarized in Table 3.

**Figure 2:**
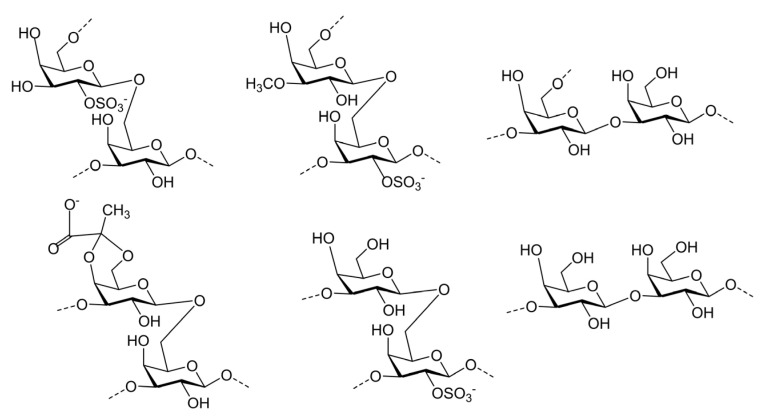
Proposed structures of the major disaccharide motifs in SGC.

The glycosidic sequence of SGC was elucidated through ^1^H–^1^H NOESY analysis. The cross signal H-1 (A)/H-6 (D) confirmed that the C-1 of the →6)-β-d-Gal*p*(2SO_4_)-(1→ residue was linked to the *O*-6 of the →3,6)-β-d-Gal*p*(2SO_4_)-(1→ residue, thereby establishing the sequence →6)-β-d-Gal*p*(2SO_4_)-(1→3,6)-β-d-Gal*p*-(1→. Correspondingly, the cross signals H-1 (C)/H-6 (B), H-1 (D)/H-3 (E), H-1(F)/H-6(D) H-1(E)/H-3(B)and H-1(E)/H-3(E) confirmed the presence of the sequences →6)-β-d-Gal*p*(3OMe)-(1→3,6)-β- d-Gal*p*(2SO_4_)-(1→, →3,6)-β-d-Gal*p*-(1→3)-β-d-Gal*p*-(1→, →3)-β-d-Gal*p*(4,6-Pyr)-(1→3,6) -β-d-Gal*p*-(1→, →3)-β-d-Gal*p*-(1→3,6) -β-d-Gal*p*(2SO_4_)-(1→ and →3)-β-d-Gal*p*-(1→3)- β-d-Gal*p*-(1→. Moreover, the cross signals H-1 (A)/H-6 (D), H-1 (C)/H-6 (B) and H-1(F)/H-6(D) demonstrated the branch features. It was speculated that the linkages →3,6)-β-d-Gal*p*-(1→3)-β-d-Gal*p*-(1→, →3)-β-d-Gal*p*-(1→3,6)-β-d-Gal*p*(2SO_4_)-(1→ and →3)-β-d-Gal*p*-(1→3)-β-D-Gal*p*-(1→ were located at the main chain. The linkages →6)-β-d-Gal*p*(2SO_4_)-(1→, →6)-β-d-Gal*p*(3OMe)-(1→ and →3)-β-d-Gal*p*(4,6-Pyr)-(1→ could be in the side chain. The primary disaccharide constituents of SGC are shown in Figure 2.

The above data revealed that SGC was a novel sulfated galactan. The backbone of SGC consisted of →3)-β-D-Gal*p*-(1→ residues, with a branch at the C-6 and partial sulfation at the C-2. The branches were composed of →3)-β-d-Gal*p*(4,6-Pyr)-(1→ and →6)-β-d-Gal*p*-(1→ unit with 2-*O*-sulfation or 3-*O*-methylation.

So far, the structural characterization of polysaccharides from the *Caulerpa* species remains poorly documented. The sulfated heteropolysaccharide (CRVP-1) from *C. racemosa* var peltata had a backbone comprising predominantly →6)-α-d-Man*p*-(1→ unit interspersed with →4)-α-d-Man*p*-(1→ and →2)-α-d-Man*p*-(1→ units. The side chain was substituted with a linear →4)-β-d-Gal*p*-(1→ unit [13]. The sulfated heteropolysaccharide (CLSP-2) from *C. lentillifera* was composed of a backbone of →6)-β-d-Man*p*-(1→ residues substituted at the *O*-2 position with sulfated side chains. The branches predominantly consisted of →3)-β-Gal*p*(4SO_4_)-(1→ and →3)-β-Gal*p*(2,4SO_4_)-(1→ residues, with trace amounts of terminal xylose units [14]. The sulfated heteropolysaccharide (F3) from *C. racemosa* contained a backbone comprising →3)-β-d-Gal*p*-(1→ residues, interspersed with terminal Xyl*p* units and →4)-Xyl*p*-(1→ branches. Additionally, Ara*f* residues were identified as (1→4)-linked and (1→3,4)-linked sequences. Sulfation occurred predominantly at the *O*-3 position of (1→4)-linked α-l-Ara*f* residues and the *O*-6 position of →3)-β-d-Gal*p*-(1→ units [26].

Moreover, it was noted that SGC exhibited distinct structural motifs compared with the pyruvylated and sulfated polysaccharides from algae. For the pyruvylated and sulfated polysaccharides from red algae, the pyruvyl groups predominantly formed six-membered cyclic ketal with *O*-4 and *O*-6 of β-d-Gal*p* residues, while sulfate substitutions were localized at the *O*-2 and *O*-4 positions of the Gal*p* units [23]. The pyruvylated and sulfated polysaccharides from the *Codium* species were isolated, and the pyruvate ketals formed cyclic acetals at either *O*-3/*O*-4 of β-d-Gal*p*-(1→ residues or *O*-4/*O*-6 of →3)-β-d-Gal*p*-(1→ units. Sulfate substitutions predominantly occupied the *O*-4 and *O*-6 positions of β-d-Gal*p* moieties [22]. Recently, a pyruvylated and sulfated galactan (PSG) from the green alga *Dictyosphaeria cavernosa* was characterized. The backbone of PSG consisted of →3)-β-d-Gal*p*-(1→ residues with partial sulfation at *O*-4 and *O*-6. Pyruvate groups formed cyclic ketals at *O*-3/*O*-4 of non-reducing terminal β-d-Gal*p* units and *O*-4/*O*-6 of internal →3)-β-d-Gal*p*-(1→ residues [23]. In comparison with the pyruvylated and sulfated polysaccharides from marine algae, the polysaccharide SGC from *C. taxifolia* featured six-membered cyclic pyruvate ketal with *O*-4 and *O*-6 of →3)-β-d-Gal*p*-(1→ residues. Additionally, SGC contained distinct structural motifs, including →6)-β-d-Gal*p*(2SO_4_)-(1→, →3,6)-β-d-Gal*p*(2SO_4_)-(1→, →6)-β-d-Gal*p* (3OMe)-(1→, →3,6)-β-d-Gal*p*-(1→, →3)-β-d-Gal*p*-(1→ and →3)-β-d-Gal*p* (4,6-Pyr)-(1→ units. These results revealed that green algae could be a potential source of unique sulfated polysaccharides.

### 2.2. Influence of SGC on α-Amylase Activity In Vitro

**Figure 3:**
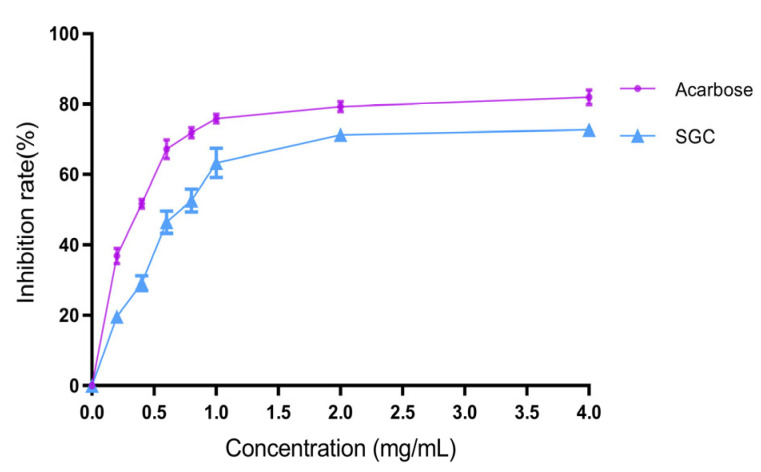
Inhibition effect of SGC on α-amylase activity.

α-Amylase is a glycoside hydrolase found in the salivary glands and pancreas that can hydrolyze starches, thus directly influencing postprandial blood glucose levels. The inhibition of α-amylase activity can delay starch digestion and modulate postprandial glycemic response. Here, the inhibition effect on α-amylase activity was assayed to evaluate the hypoglycemic activity of SGC in vitro. As shown in Figure 3, SGC exhibited a significant inhibitory effect of α-amylase activity in a concentration-dependent pattern. At the concentration of 4 mg/mL, the inhibition rate of SGC on α-amylase activity was about 72.7%. The IC_50_ values of acarbose and SGC were 0.437 ± 0.075 and 0.608 ± 0.057 mg/mL, respectively. The inhibition effect of α-amylase activity by SGC was slightly lower than that by acarbose. Therefore, SGC had prospects as a hypoglycemic agent. Similarly, the sulfated polysaccharides from *Caulerpa lentillifera* exhibited potent α-glucosidase inhibitory activity (IC_50_ = 134.81 ± 2.0 µg/mL), demonstrating their potential as an anti-diabetic therapeutic [27]. The anti-diabetic activity of SGC in vivo was further investigated using T2DM mice.

### 2.3. Anti-Diabetic Activity of SGC In Vivo

Streptozotocin (STZ) can kill a portion of islet β-cells, and high-fat diet may cause an increase in blood glucose and serum lipid metabolic disorders, as well as insulin resistance. Thus, the combination of high-fat diet and low-dose STZ is often used as a T2DM inducer for experimental T2DM mice model. In the present study, the T2DM mouse models induced by a combination of high-fat diet and low-dose STZ was established for the study of anti-diabetic activity of SGC in vivo.

#### 2.3.1. Influences of SGC on Body Weight and Fasting Blood Glucose Level

**Figure 4:**
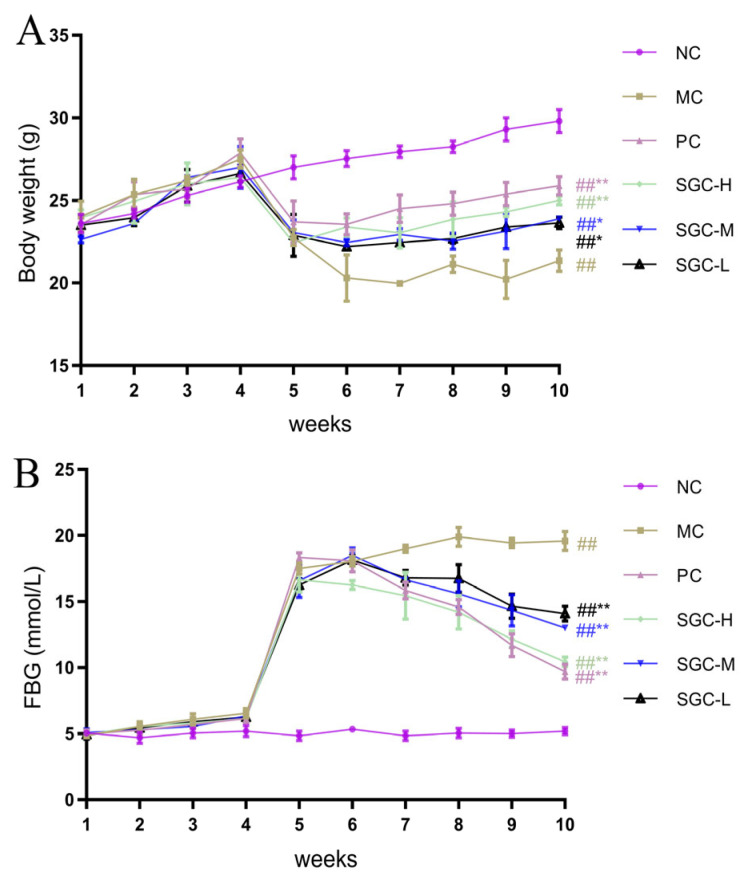
Effects of SGC on body weight and FBG in T2DM mice. (**A**) Body weight and (**B**) FBG levels. NC: the normal control group received standard chow; MC: the diabetic model control group; PC: the positive control group treated with metformin at the dose of 200 mg/kg/day; SGC-L: the low-dose SGC group treated with SGC at the dose of 100 mg/kg/day; SGC-M: the middle-dose SGC group treated with SGC at the dose of 200 mg/kg/day; SGC-H: the high-dose SGC group treated with SGC at the dose of 400 mg/kg/day. Data were expressed as the mean ± standard deviation (SD) (*n* = 8). Inter-group comparisons utilized one-way analysis of variance (ANOVA) followed by Tukey’s post hoc test. ^##^ *p* < 0.01 versus NC group, * *p* < 0.05 and ** *p* < 0.01 versus MC group.

The modulatory effect of SGC on body weight in the T2DM mice is illustrated in Figure 4A. Following a 7-day acclimatization period, all groups showed an increase in body weight. During the second to fourth weeks, the body weights in the normal control (NC) group and the model control (MC) group continued to increase, especially the MC group. This phenomenon is likely attributable to lipid deposition induced by the high-fat diet. A critical transition occurred in the fifth week. Intraperitoneal injection of STZ induced an obvious loss of body weight in the MC group, consistent with the pathological feature of diabetic wasting syndrome. Despite the commencement of metformin and SGC interventions in the sixth week, all groups still displayed a slight decrease in body mass, indicating the persistent cytotoxic effects of STZ on pancreatic β-cells. However, in the seventh week, the SGC intervention groups exhibited a dose-dependent trend toward the recovery of body weight. In the tenth week, the body weight gain in the high dose of SGC (SGC-H) group was significantly higher (3.8 g, *p* < 0.01) than that in the MC group, accounting for 80.9% of the weight gain observed in the positive control (PC) group. Compared with the MC group, T2DM mice in the low dose of SGC (SGC-L) and middle dose of SGC (SGC-M) groups also demonstrated a significant capacity for the restoring of body weight (*p* < 0.05). These data indicate that SGC reversed the energy metabolism imbalance in the T2DM mice and ameliorated diabetes-associated weight abnormalities.

Following food intake, digestion and absorption, an organism undergoes a series of changes due to elevated blood glucose levels. Persistent hyperglycemia triggers oxidative stress in the body and leads to functional impairment of pancreatic β-cells. Therefore, controlling fasting blood glucose (FBG) is a critical objective of treating diabetes. In T2DM mice, FBG serves as an important indicator of blood glucose balance. The influence of SGC on the level of FBG was assayed using the T2DM mice, and the result is shown in Figure 4B. During the therapeutic intervention period of five weeks, the three SGC groups exhibited divergent trends in FBG. In the sixth week, the PC and SGC-H groups demonstrated a declining trend in FBG, while the SGC-M and SGC-L groups still showed a slight upward trend. From the seventh to tenth weeks, the levels of FBG in the PC group and the three SGC groups exhibited a sustained downward trend. These results demonstrate that SGC significantly modulated FBG levels compared with the MC group (*p* < 0.01) and ameliorated glucose metabolism disorders.

#### 2.3.2. Effect of SGC on Glucose Tolerance

Blood glucose measurement is a key diagnostic indicator for diabetes. However, due to the influence of various factors, dynamic fluctuations and individual variations in blood glucose levels, false-positive readings may occur [28]. Therefore, the oral glucose tolerance test (OGTT) is employed for diabetes diagnosis [29,30].

**Figure 5:**
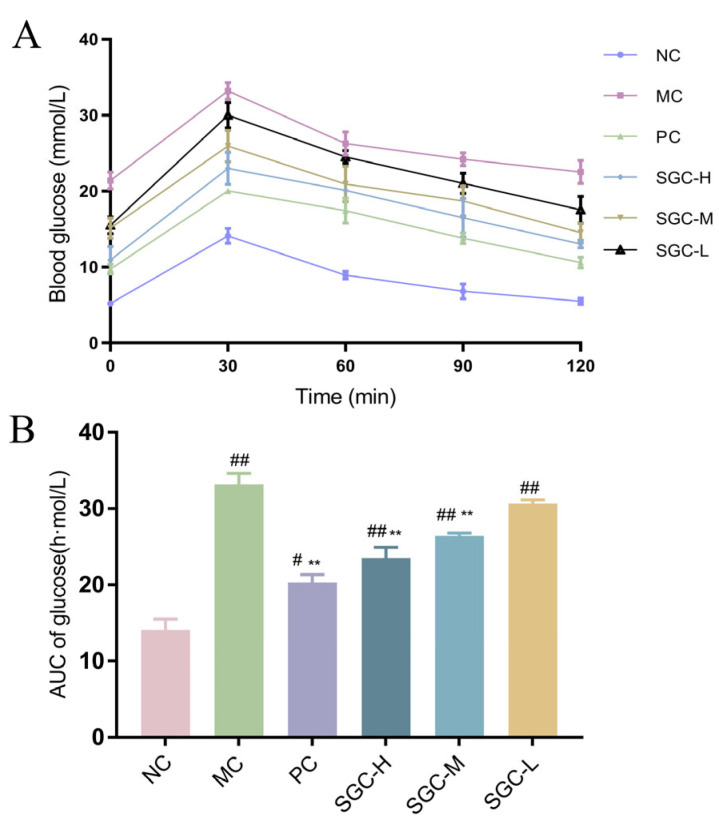
Effects of SGC on OGTT and OGTT-AUG in the T2DM mice. (**A**) OGTT and (**B**) OGTT-AUG. NC: the normal control group received standard chow; MC: the diabetic model control group; PC: the positive control group treated with metformin at the dose of 200 mg/kg/day; SGC-L: the low-dose SGC group treated with SGC at the dose of 100 mg/kg/day; SGC-M: the middle-dose SGC group treated with SGC at the dose of 200 mg/kg/day; SGC-H: the high-dose SGC group treated with SGC at the dose of 400 mg/kg/day. Data were expressed as the mean ± SD (*n* = 8). Inter-group comparisons utilized one-way ANOVA followed by Tukey’s post hoc test. ^#^ *p* < 0.05 and ^##^ *p* < 0.01 versus NC group, ** *p* < 0.01 versus MC group.

As shown in Figure 5A, all groups exhibited characteristic dynamic blood glucose curves following glucose administration. Peak values of blood glucose levels were observed at 30 min post-gavage. The maximum value of blood glucose in the NC group was 13.6 mM, while the MC group showed a significant elevation to 33.2 mM, confirming functional impairment of pancreatic β-cells. At 120 min post-glucose administration, the NC group returned to the baseline value of blood glucose levels, whereas the MC group remained hyperglycemic. These data demonstrated that glucose tolerance was damaged critically in the T2DM mice. However, compared with the MC group, SGC groups exhibited dose-dependent improvement, especially the SGC-H group. The glucose level in the SGC-H group decreased to 13.05 mM, but remained 23.1% higher than that in the PC group, indicating that the certain dose of SGC could ameliorate glucose tolerance. In addition, the three SGC groups showed a reduction in the area under the oral glucose tolerance test curve (OGTT-AUC) (Figure 5B), indicating that SGC effectively improved glucose metabolism. These data further demonstrated that SGC possessed significant glucose-regulating effects.

#### 2.3.3. Effect of SGC on Insulin Resistance

Insulin critically regulates glucose homeostasis and lipid metabolism. Prolonged hyperglycemia and elevated free fatty acids induce metabolic dysregulation, impairing insulin signaling pathways and consequently triggering insulin resistance [31]. Several cost-effective, minimally invasive methods quantify insulin resistance. The quantitative insulin sensitivity check index (QUICKI), the index of homeostasis model assessment of insulin resistance (HOMA-IR) and the index of homeostasis model assessment β (HOMA-β) are calculated from fasting glucose and insulin levels [32]. These clinically established metrics provide standardized assessments of insulin resistance. In the present study, the assays of the fasting insulin content, HOMA-IR, HOMA-β and QUICKI were used for the evaluation of insulin resistance.

As shown in Table 4, compared with the NC group, fasting insulin levels and HOMA-IR index in the MC group were markedly elevated, whereas QUICKI and HOMA-β indexes were apparently reduced, illustrating that the T2DM mice had insulin resistance. Compared with the MC group, fasting insulin content significantly decreased in the SGC-H group (*p* < 0.01), while the SGC-M and SGC-L groups showed a decreasing trend without reaching statistical significance. Additionally, SGC reduced the HOMA-IR index in a dose-dependent manner compared with the MC group. After five weeks of continuous administration, compared with the MC group, the QUICKI and HOMA-β indexes significantly increased in the SGC-H group (*p* < 0.01), while the increase in the SGC-M and SGC-L groups showed no significant difference. The levels of the fasting insulin content, HOMA-IR and QUICKI indexes in the SGC-H group were close to those in the PC group. These results suggested that SGC effectively regulated insulin homeostasis and mitigates insulin resistance in the T2DM mice.

#### 2.3.4. Effect of SGC on Serum Lipid

**Figure 6:**
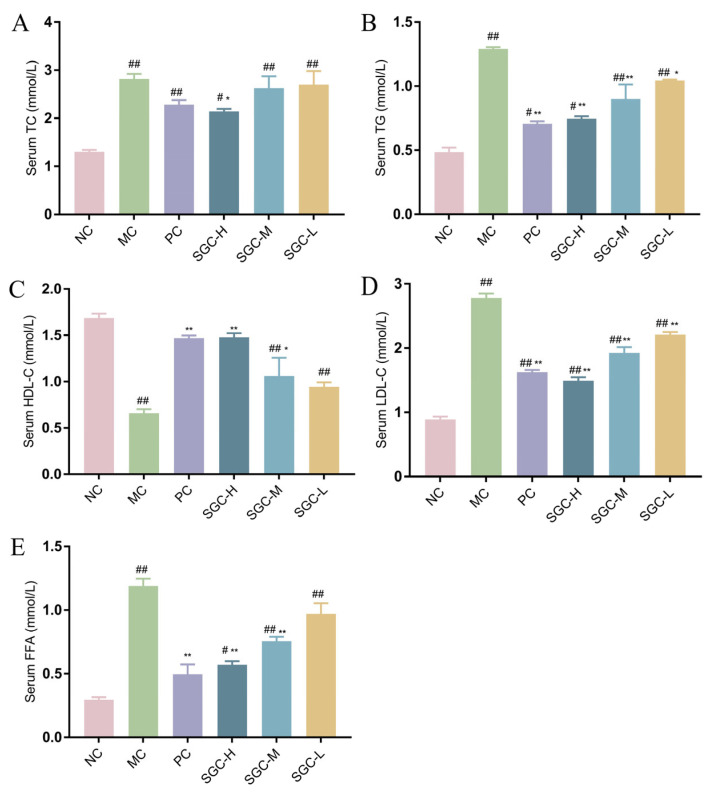
Effect of SGC on serum lipid in the T2DM mice. (**A**) TC level; (**B**) TG level; (**C**) HDL-C level; (**D**) LDL-C level; and (**E**) FFA level. NC: the normal control group received standard chow; MC: the diabetic model control group; PC: the positive control group treated with metformin at the dose of 200 mg/kg/day; SGC-L: the low-dose SGC group treated with SGC at the dose of 100 mg/kg/day; SGC-M: the middle-dose SGC group treated with SGC at the dose of 200 mg/kg/day; SGC-H: the high-dose SGC group treated with SGC at the dose of 400 mg/kg/day. Data were expressed as the mean ± SD (*n* = 8). Inter-group comparisons utilized one-way ANOVA followed by Tukey’s post hoc test. ^#^ *p* < 0.05 and ^##^ *p* < 0.01 versus NC group, * *p* < 0.05 and ** *p* < 0.01 versus MC group.

The therapeutic effect of SGC on T2DM can be evaluated through its impact on lipid metabolism [33,34]. By comparison with the NC group, the levels of triglycerides (TG), total cholesterol (TC), low-density lipoprotein cholesterol (LDL-C) and free fatty acid (FFA) were obviously elevated in the MC group, and the amount of high-density lipoprotein cholesterol (HDL-C) were largely reduced (Figure 6A–E). It was observed that after treatment for five weeks, the three SGC groups demonstrated significant reductions in TG, TC, LDL-C and FFA levels and an increase in the HDL-C level compared with the MC group. Moreover, the effect of SGC on TG, TC, LDL-C, FFA and HDL-C levels occurred in a dose-dependent manner. Additionally, the effects of SGC on TC, LDL-C and HDL-C levels in the SGC-H group were stronger than those of the positive control, and the effects of SGC on TG and FFA levels were slightly weaker. These data confirmed that SGC improved dyslipidemic abnormalities and possessed therapeutic potential for lipid homeostasis modulation.

#### 2.3.5. Effect of SGC on Oxidative Stress

Patients with T2DM exhibit a state of oxidative stress characterized by significantly elevated oxidative stress markers in serum, leukocytes and pancreatic tissues. The superoxide dismutase (SOD), glutathione (GSH), malonaldehyde (MDA) and catalase (CAT) are commonly measured to assess oxidative stress levels [6,11]. Oxidative stress is linked to pancreatic β-cell dysfunction, insulin resistance and diabetic complications, highlighting the critical role of antioxidant therapy in diabetes prevention and management.

As shown in Table 5, the MC group exhibited a characteristic oxidative stress profile compared with the NC group. This characteristic oxidative stress was manifested as significantly diminished levels of the key antioxidant enzymes SOD, CAT and GSH in the MC group. Concomitantly, the MC group displayed a marked elevation in MDA, a reliable biomarker of lipid peroxidation resulting from oxidative damage. However, after treatment with SGC, this oxidative imbalance noticeably improved in the SGC-H group compared with the MC group, but the levels of SOD, CAT, GSH and MDA were not significantly different in the SGC-L group. Thus, SGC demonstrated a dose-dependent antioxidant effect, and the most significant restoration of mitigation of oxidative stress was achieved in the SGC-H group. In addition, it was obvious that metformin treatment induced a significant increase in the levels of SOD, CAT and GSH, and resulted in a decrease in the MDA level in comparison with the MC group. The levels of SOD, CAT and GSH in the SGC-H group were slightly lower than those of metformin, whereas the level of MDA was slightly higher. These pronounced changes indicated a substantial restoration of antioxidant defense capacity and attenuation of oxidative damage in the T2DM mice.

#### 2.3.6. Histopathological Analysis of Liver and Pancreas

**Figure 7:**
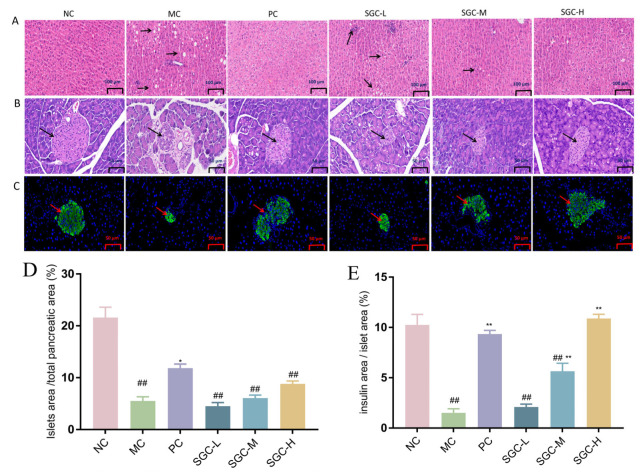
Histological analyses of liver and pancreas in the T2DM mice. (**A**) Representative images of liver from sections stained with H&E; (**B**) representative images of pancreas from sections stained with H&E; (**C**) representative images of pancreatic β cell from sections stained with immunofluorescence; (**D**) quantification of islet area; and (**E**) the ratios of β-cell area to total islet area. Arrows: areas with differences. NC: the normal control group received standard chow; MC: the diabetic model control group; PC: the positive control group treated with metformin at the dose of 200 mg/kg/day; SGC-L: the low-dose SGC group treated with SGC at the dose of 100 mg/kg/day; SGC-M: the middle-dose SGC group treated with SGC at the dose of 200 mg/kg/day; SGC-H: the high-dose SGC group treated with SGC at the dose of 400 mg/kg/day. Data were expressed as the mean ± SD (*n* = 8). Inter-group comparisons utilized one-way ANOVA followed by Tukey’s post hoc test. ^##^ *p* < 0.01 versus NC group, * *p* < 0.05 and ** *p* < 0.01 versus MC group.

To evaluate the potential impact of SGC on the liver and pancreas, histopathological analyses of the liver and pancreas were performed in the T2DM mice. It is established that a high-fat diet combined with STZ can induce hepatic inflammation, necrosis and fibrosis and reduce islet mass [35]. Hematoxylin and eosin (H&E) staining was utilized to assess histopathological alterations in hepatic and pancreatic tissues of the T2DM mice [36]. As illustrated in Figure 7A, the NC group exhibited intact hepatic architecture with well-organized cell alignment. In contrast, disorganized hepatocyte arrangement and vacuolation were observed in the MC group. The SGC-H group demonstrated a significant reduction in vacuolation, decrease in hepatocyte gaps and improvement in cellular morphology compared with the MC group. The ameliorative effects were comparable to those observed in the PC group. Both the SGC-M and SGC-L groups showed enhanced uniformity in hepatocyte arrangement, and the effects were dose-dependent.

Distinct pancreatic morphological alterations were observed across experimental groups (Figure 7B). The ratio of islet area to pancreatic area was increased by SGC administration in a dose-dependent manner (Figure 7D). Well-organized pancreatic cell arrangement, clearly defined islet boundaries and abundant islet cell numbers were observed in the NC group. Conversely, disorganized pancreatic architecture, fragmented and irregular islets, significantly depleted islet cells and indistinct islet boundaries were evident in the MC group. Compared with the MC group, dose-dependent restorative effects on pancreatic tissue in the T2DM mice were observed by SGC administration. Progressive islet enlargement, increased islet cell repopulation and enhanced definition of islet boundaries occurred with escalating SGC doses. These results indicated that SGC effectively repaired pancreatic damage in the T2DM mice, with the most pronounced therapeutic efficacy observed in the SGC-H group.

Immunofluorescence analysis of pancreatic insulin reveals that insulin is predominantly localized in the central islet region, with green fluorescence intensity exhibiting a positive correlation with insulin content and β-cell abundance [37]. Compared with the NC group, the insulin-positive β-cell area was significantly reduced in the MC group (Figure 7C,E), suggesting suppressed insulin expression and substantial β-cell depletion. The insulin-positive area was restored by SGC, indicating enhanced insulin secretion and recovered islet functionality. Compared with the MC group, the SGC-H group exhibited the most pronounced ameliorative effect (*p* < 0.01), which was approaching that of the PC group. These results demonstrated that SGC exhibited significant therapeutic efficacy in ameliorating histopathological lesions in both hepatic and pancreatic tissues of the T2DM mice.

Taken together, SGC possessed a remarkable anti-diabetic activity in vivo. SGC significantly improved blood glucose balance and glucose tolerance, ameliorated insulin resistance and enhanced insulin sensitivity. SGC also markedly reduced the levels of the serum TC, TG, LDL-C and FFA and increased HDL-C content, demonstrating regulatory effects on lipid metabolism disorders. Furthermore, SGC significantly elevated the levels of the serum SOD, CAT and GSH, and decreased MDA content, thereby alleviating systemic oxidative stress injury. Additionally, SGC ameliorated histopathological alterations in the liver and pancreas and restored insulin secretion in pancreatic β-cells. The most characteristic feature of T2DM is hyperglycemia, which may induce oxidative stress and lead to systemic dysfunction. Blood glucose regulation is closely linked to insulin secretion and action, thus controlling blood glucose and modulating insulin secretion represent two key therapeutic approaches for T2DM [33]. Oxidative stress is associated with pancreatic β-cell function, insulin resistance and diabetic complications; antioxidant capacity plays a critical role in diabetes prevention and management. PCR ameliorated hyperglycemia, dyslipidemia, and renal oxidative stress, thereby exhibiting anti-diabetic and nephroprotective effects by impeding oxidative stress and inflammation [17]. The polysaccharide from *Caulerpa lentillifera* waste, which demonstrated antioxidant and α-glucosidase inhibitory activity, shows potential as a food supplement for diabetes prevention [38]. The present results revealed that SGC mitigated oxidative stress and insulin resistance and improved glucose homeostasis and lipid metabolism. SGC has the potential to be developed into a novel anti-diabetic agent. Continued work on the pharmacokinetics and toxicity of SGC is needed.

Current evidence indicates a strong association between the anti-diabetic and antioxidant activities of green algal polysaccharides, though studies reporting this dual activity remain limited. *Enteromorpha prolifera* polysaccharide (EPP) contained α-l-Rha*p*-(1→, →4)-α-l-Ara*p*-(1→, →2)-α-l-Rha*p*-(1→, →3)-β-d-Gal*p*-(1→ and →3)-β-d-Glc*p*A-(1→ units. EPP exerted excellent hypoglycemic effects through enhanced antioxidant capacity and reduced lipid peroxidation damage in vivo [30]. *Ulva lactuca* polysaccharide (ULP) was mainly composed of →2,3)-α-l-Rha*p*-(1→, →4)-β-d-Glc*p*A-(1→, →2,6)-β-d-Gal*p*-(1→ and →4)-β-d-Xyl*p*-(1→ units. ULP alleviated T2DM by improving insulin tolerance, increasing SOD and CAT activities and thus lowering blood glucose level [39]. At the same concentration, SGC was superior to EPP in both OGTT and antioxidant activity and to UPP in alleviating insulin resistance. However, definitive structure–activity relationships for the anti-diabetic and antioxidant activities of these green algal polysaccharides cannot yet be established. The universality of these correlations must be further investigated.

## 3. Materials and Methods

### 3.1. Materials

*C. taxifolia* was collected from the coastal waters of Xuwen County in Zhanjiang, Guangdong Province, China in March 2021. The raw alga was thoroughly rinsed with tap water, followed by three washes with distilled water. It was then air-dried (20–25 °C) milled and stored in a controlled dry environment. Sephacryl S-400/HR and Q Sepharose Fast Flow were from GE Healthcare Life Sciences (Piscataway, NJ, USA). α-Amylase were from Beijing Solarbio Science & Technology Co., Ltd. (Beijing, China). TG, TC, LDL-C, HDL-C, FFA, SOD, GSH, MDA and CAT assay kits were from Nanjing Jiancheng Bioengineering Institute (Nanjing, China). STZ were from Aladdin Chemical Co., Ltd. (Shanghai, China).

### 3.2. Animals

The C57BL/6J mice (male, 23 ± 2 g, 8 weeks) were from Jinan Pengyue Laboratory Animal Breeding Co., Ltd. (Jinan, Shandong, China). The animals were maintained under controlled environmental conditions (23 ± 2 °C; 12 h light/dark cycle) with ad libitum access to standard chow and purified water.

### 3.3. Extraction and Isolation of SGC

The extraction and isolation protocol of the polysaccharide were adapted from established procedures reported in previous studies [23]. Briefly, the milled seaweed (500 g) was extracted with a 40-fold volume of deionized water at 100 °C for 4 h. After filtration, the residue was extracted using 0.5 M NaOH (4 L) for 4 h at room temperature. After centrifugation (5000× *g*, 10 min), the supernatant was precipitated by adding 95% ethanol (4 vols), which produced a crude polysaccharide (38.4 g). The resulting product was subsequently fractionated on a Q Sepharose Fast Flow column (5.0 cm × 50 cm) eluted with a stepwise NaCl gradient (0, 0.5, 1.0, 1.5 and 2 M). The 0.5 M NaCl-eluted fraction was collected, desalted and further purified using a Sephacryl S-400/HR column (2.5 cm × 90 cm) with 0.2 M NH_4_HCO_3_ as the mobile phase. The final purified polysaccharide was designated as SGC (7.8 g).

### 3.4. Assay of Physicochemical Property

Total sugar content was determined by the phenol-sulfuric acid method [40]. Sulfate content was analyzed by the BaCl_2_-gelatin method [41]. Uronic acid was assayed using the carbazole-sulfuric acid method [42]. Protein was measured as described by the Bicinchoninic Acid (BCA) protein assay reagent kit. Pyruvic acid was quantified following the procedure established by the 2,4-dinitrophenylhydrazine method [43]. Purity assessment and molecular mass determination were executed through HPGPC [44]. Chromatographic analysis was conducted using an Agilent 1260 Infinity HPLC instrument (Agilent Technologies, Santa Clara, CA, USA) fitted with a Shodex OHpak SB-804 HQ column (8.0 mm× 300 mm, Showa Denko K.K., Tokyo, Japan) and an Agilent RID-10A (Agilent Technologies, Santa Clara, CA, USA) refractive index detector. Molecular weight estimation employed a calibration curve generated from pullulan standards (Mw: 4.6, 5.9, 9.6, 21.1, 47.1, 107, 200, 344 and 708 kDa, 99% purity, Showa Denko K. K., Tokyo, Japan).

Monosaccharide composition was analyzed by HPLC [45]. Briefly, polysaccharide was hydrolyzed with 2 M trifluoroacetic acid at 110 °C for 4 h, derived with 1-phenyl-3-methyl-5-pyrazolone and assayed on an Agilent 1260 Infinity HPLC instrument fitted with an Agilent XDB-UV detector (254 nm) and an Eclipse XDB-C_18_ column (4.6 mm× 250 mm, Agilent Technologies, Santa Clara, CA, USA), using 0.1 M KH_2_PO_4_ and acetonitrile (84:16 *v*/*v*) as eluent at 1.0 mL/min and 35 °C. Identification of sugar was performed by comparison with retention time of reference sugars (d-mannose, d-glucosamine, l-rhamnose, d-glucuronic acid, d-galacturonic acid, d-glucose, d-galactose, d-xylose, l-arabinose and l-fucose, 99% purity, Sigma, St. Louis, MO, USA, 99% purity, Sigma, St. Louis, MO, USA).

Sugar configuration was analyzed by reversed-phase HPLC [21]. Polysaccharide was degraded with 2 M trifluoroacetic acid at 110 °C for 4 h, then reacted with l-cysteine methyl ester in pyridine (60 °C, 1 h). After adding o-tolyl isothiocyanate, the mixture was heated (60 °C, 1 h). The reaction mixture was analyzed on an Agilent 1260 Infinity HPLC instrument fitted an Eclipse XDB-C_18_ column (4.6 mm × 250 mm) and an Agilent XDB-UV detector (250 nm), using deionized water and acetonitrile (80:20 *v*/*v*) as eluent at 0.8 mL/min and 35 °C.

Desulfation of SGC was performed via dimethyl sulfoxide (DMSO) solvolysis, followed by additional desulfation using the benzene-1,2,4,5-tetracarboxylic acid method [46], yielding the final product dsSGC. Pyruvic acid residues were eliminated from SGC and dsSGC following the previous method [23]. Briefly, 20 mg of sample was dissolved in 10 mL of 1% (*v*/*v*) aqueous acetic acid at 100 °C for 6 h. The reaction mixture was neutralized with sodium bicarbonate, dialyzed against distilled water and lyophilized, yielding the depyruvylated derivatives dpSGC and dsdpSGC, respectively.

### 3.5. Methylation Analysis

The experimental procedure was conducted in accordance with the established method by Harris et al. [47]. Briefly, polysaccharide was dissolved in DMSO and subject to sequential methylation using NaH and CH_3_I. The methylated product was then hydrolyzed with 2 M trifluoroacetic acid at 105 °C for 6 h. Subsequently, the hydrolysate was reduced with NaBD_4_ and acetylated with acetic anhydride in the presence of pyridine, yielding partially methylated alditol acetates. Gas chromatography–mass spectrometry analysis was performed on a TRACE 1300-ISQ instrument (Thermo Scientific, Waltham, MA, USA) equipped with a DB-225 fused silica capillary column (0.25 mm × 30 m, 0.25 µm, Agilent Technologies, Santa Clara, CA, USA).

### 3.6. NMR Spectroscopy

The sample (50 mg) was subjected to deuterium exchange through multiple cycles of lyophilization in 99.9% D_2_O and subsequently resuspended in D_2_O for analysis. One-dimensional and two dimensional NMR spectra were acquired at 25 °C using an Agilent DD2 500 MHz NMR spectrometer (Agilent Technologies Co. Ltd., Palo Alto, CA, USA). Chemical shifts were referenced to acetone (δ_H_ 2.225, δ_C_ 31.07), which served as the internal reference standard. Spectra were processed and analyzed using MestReNova (V12.0.3, Mestrelab Research, Spain).

### 3.7. α-Amylase Inhibitory Assay

The inhibitory activity of polysaccharides against α-amylase was quantified using a modified 3,5-dinitrosalicylic acid method [48]. Porcine pancreatic α-amylase was diluted to 0.5 U/mL using sodium phosphate buffer (pH 6.9). Test polysaccharide and acarbose (positive control) were prepared in graded concentrations (0, 0.2, 0.4, 0.6, 0.8, 1.0, 2.0 and 4.0 mg/mL) with the same buffer. Reaction systems containing 200 μL polysaccharide or acarbose solution and 200 μL enzyme solution were homogenized and incubated at 37 °C for 10 min. Subsequently, 1 mL soluble starch solution (1% *w*/*v*) was added to each tube, followed by additional incubation for 10 min at 37 °C. The enzymatic reaction was terminated by adding 1 mL DNS reagent and boiling for 10 min. After cooling, samples were diluted with deionized water and absorbance measured at 540 nm.

Inhibition rate (%) = (1 − (A1 − A2)/(A3 − A4)) × 100%

A1: Test compound + α-amylase (inhibition group); A2: test compound without α-amylase (inhibition control); A3: α-Amylase without test compound (enzyme blank); A4: buffer only (reagent blank)

### 3.8. Animal Experiment

C57BL/6J mice (*n* = 48, male, 23 ± 2 g) were housed under specific pathogen-free conditions. After one week of acclimatization, the body weights and FBG levels of the mice were measured. Eight mice were randomly divided as the NC group and received standard chow. The other mice were fed a high-fat/high-sucrose diet (59% basal feed, 18% lard, 3% cholesterol and 20% sucrose) for three weeks and then received intraperitoneal injections of STZ (30 mg/kg/day) for three consecutive days. The FBG levels of the mice were assessed 72 h after the final injection. The mice with FBG ≥ 11.1 mM were classified as T2DM model mice [31] and randomly divided into five groups (*n* = 8 per group): MC group, PC group and three SGC groups (SGC-L, SGC-M and SGC-H). The mice in the SGC-L, SGC-M and SGC-H groups were treated with SGC at a dose of 100, 200, or 400 mg/kg/day, respectively, by intragastric administration. Here, the concentrations of SGC were chosen on the basis of a pre-experiment and the related literature [5]. The mice in the PC group were treated with metformin at a dose of 200 mg/kg/day, whereas the mice in the NC and MC groups received equivalent volumes of 0.9% saline solution. All treatments were delivered daily via intragastric gavage. The body weights and FBG levels of mice were monitored weekly. Following a 5-week treatment period, the mice were fasted for 12 h with free access to water, followed by blood collection via retro-orbital sinus puncture. The serum was isolated by centrifugation at 2000× *g* for 15 min at 4 °C and stored at −80 °C until analysis. The mice were euthanized by cervical dislocation, and the liver and pancreatic tissues were immediately excised and fixed in 4% paraformaldehyde for histopathological analysis. All experimental procedures were conducted in accordance with protocols approved by the institutional animal care and use committee of Ocean University of China (OUC-SMP-2024-04-02).

### 3.9. Analyses of Glucose and Insulin Tolerance

OGTT, HOMA-IR, HOMA-β and QUICKI were calculated to evaluate the sensitivity of insulin. After 5 weeks of oral gavage administration, mice were food-deprived for 12 h with free access to water, followed by oral administration of 2 g/kg glucose solution. Blood samples were collected from the tail vein at 0, 30, 60, 90 and 120 min post-administration for blood glucose measurement. OGTT-AUC was calculated using the trapezoidal rule [49]. After the final administration, mice were fasted for 12 h. Blood samples were collected via the orbital sinus under anesthesia, followed by centrifugation at 2000× *g* for 15 min to isolate serum. Fasting serum insulin (FINS) levels were quantified using a mouse insulin ELISA kit according to the manufacturer’s instructions. The HOMA-IR, HOMA-β and QUICKI were calculated as follows [32,50,51,52]:

HOMA−IR = [FBG (mM) × FISN (μU/mL)]/22.5, HOMA-β = [20 × FISN (μU/mL)]/[FBG (mM)−3.5] and QUICKI = 1/[*log* FINS (μU/mL) + *log* FBG (mM)].

### 3.10. Determination of Lipid Metabolic Parameters

The contents of the serum TG, TC, HDL-C, LDL-C and FFA were determined according to the kit instructions.

### 3.11. Measurement of Oxidative Stress Parameters

The levels of the serum SOD, MDA, CAT and GSH were measured using the assay kits.

### 3.12. Histopathological Analysis and Immunofluorescence Staining

Liver and pancreatic tissues were fixed in 4% paraformaldehyde, embedded in paraffin and sectioned into 5 μm thick slices using a microtome. For histopathological evaluation, tissue sections were stained with H&E and analyzed under a light microscope (VS200; Olympus, Tokyo, Japan). Immunofluorescence staining was performed with goat-derived polyclonal anti-insulin antibodies, and images were captured using a laser scanning confocal microscope (Pannoramic MIDI; 3DHISTECH, Budapest, Hungary). The islet area and insulin-positive area were conducted using Image J (1.53 k) software.

### 3.13. Statistical Analysis

Data were presented as the mean ± SD. Inter-group comparisons utilized one-way ANOVA followed by Tukey’s post hoc test and non-parametric Kruskal–Wallis test with subsequent Dunn’s post hoc test. All analyses utilized GraphPad Prism 9.2.0 software, with a significance threshold of *p* < 0.05.

## 4. Conclusions

The sulfated polysaccharide SGC from the green alga *C. taxifolia* was a sulfated galactan and was substituted with pyruvic acid ketals. The backbone consisted of a →3)-β-**d**-Gal*p*-(1→ unit, with branching at the C-6 and partial sulfation at C-2. The branches were composed of →6)-β-d-Gal*p*(3OMe)-(1→, →3)-β-d-Gal*p*(4,6-Pyr)-(1→ and →6)-β-d-Gal*p*(2SO_4_)-(1→. SGC significantly improved glucose tolerance, ameliorated lipid metabolism, alleviated oxidative stress and enhanced insulin sensitivity in vivo. The effects in the high-dose SGC group were close to those observed in the positive control group. These data suggest that SGC might be a potential anti-diabetic agent. Restoring gut microbial homeostasis is an important mechanism through which dietary polysaccharides exert their beneficial effects. As SGC is a complex, macromolecular carbohydrate that is largely indigestible and poorly absorbed, its anti-diabetic effects are likely mediated indirectly via modulation of the gut microbiota [53,54]. Low-molecular-weight breakdown products of polysaccharides generated by microbiota can enter the systemic circulation [55,56], which is a process that may contribute to the anti-diabetic effects of SGC. The relationship between the anti-diabetic activity of SGC and the gut microbiota requires further investigation. Further investigation on the pharmacokinetics and toxicity of SGC will be essential to understand the results of in vivo experiments.

## Figures and Tables

**Table 1:** Methylation analysis results of SGC, dsSGC, dpSGC and dsdpSGC.

Methylated Alditol Acetate	Molar Percentage				Type of Linkage
	SGC	dsSGC	dpSGC	dsdpSGC	
1,5-Di-*O*-acetyl-2,3,4,6-tetra-*O*-methyl-galactitol	17.2	16.9	18.3	18.9	Gal*p*-(1→
1,3,5-Tri-*O*-acetyl-2,4,6-tri-*O*-methyl-galactitol	34.1	35.5	40.2	39.1	→3)-Gal*p*-(1→
1,5,6-Tri-*O*-acetyl-2,3,4-tri-*O*-methyl-galactitol	15.8	21.7	14.7	23.3	→6)-Gal*p*-(1→
1,2,5,6-Tetra-*O*-acetyl-3,4-di-*O*-methyl-galactitol	6.3	- ^a^	5.7	- ^a^	→2,6)-Gal*p*-(1→
1,3,5,6-Tetra-*O*-acetyl-2,4-di-*O*-methyl-galactitol	12.3	19.4	12.9	18.7	→3,6)-Gal*p*-(1→
1,3,4,5,6-Penta-*O*-acetyl-2-*O*-methyl-galactitol	6.1	6.5	- ^a^	- ^a^	→3,4,6)-Gal*p*-(1→
1,2,3,5,6-Penta-*O*-acetyl-4-*O*-methyl-galactitol	8.2	- ^a^	8.2	- ^a^	→2,3,6)-Gal*p*-(1→

^a^ Not detected.

**Table 2:** Signal assignments of NMR spectra of SGC.

	Sugar Residues	Chemical Shifts (δ) ^a^					
		H1/C1	H2/C2	H3/C3	H4/C4	H5/C5	H6/C6
A	→6)-β-d-Gal*p*(2SO_4_)-(1→	4.93/103.93	4.36/79.71	3.75/73.87	4.27/70.21	3.81/76.29	4.00/69.89
B	→3,6)-β-d-Gal*p*(2SO_4_)-(1→	4.87/101.89	4.36/79.71	3.95/83.51	4.27/70.21	3.81/76.29	4.05/68.39
C	→6)-β-d-Gal*p*(3OMe)-(1→	4.73/103.26	3.73/70.92	3.47/81.72	4.18/68.37	3.76/74.01	4.09/69.89
D	→3,6)-β-d-Gal*p*-(1→	4.70/104.76	3.71/71.80	3.93/83.42	4.18/68.37	3.76/74.01	3.94/69.96
E	→3)-β-d-Gal*p*-(1→	4.57/104.45	3.74/71.90	3.90/84.09	4.18/68.37	3.96/74.11	3.80/62.57
F	→3)-β-d-Gal*p*(4,6-Pyr)-(1→	4.53/104.77	3.61/72.11	4.38/77.25	4.17/72.56	3.73/67.19	3.99/66.67
G	β-d-Gal*p*-(1→	4.51/105.44	3.61/72.11	3.60/72.53	4.18/68.37	3.70/74.41	3.78/62.57

^a^ Spectra were performed on an Agilent DD2 500M NMR spectrometer. Chemical shifts are referenced to internal acetone at δH 2.225 and δC 31.07. Gal*p*: galactopyranose.

**Table 3:** Signal assignments of NMR spectra of dsSGC.

	Sugar Residues	Chemical Shifts (δ) ^a^					
		H1/C1	H2/C2	H3/C3	H4/C4	H5/C5	H6/C6
C	→6)-β-d-Gal*p*(3OMe)-(1→	4.72/105.36	3.65/71.89	3.47/81.62	4.26/69.98	3.71/74.46	3.92, 4.09/70.24
D	→3,6)-β-d-Gal*p*-(1→	4.71/104.78	3.75/71.32	3.84/83.68	4.19/70.15	3.76/76.31	3.97, 4.09/70.95
E	→3)-β-d-Gal*p*-(1→	4.55/105.70	3.72/72.72	3.89/83.52	4.16/70.14	3.94/75.51	3.82, 3.92/62.57
F	→3)-β-d-Gal*p*(4,6-Pyr)-(1→	4.52/104.33	3.60/72.23	4.29/79.75	4.20/72.44	3.67/67.42	3.98, 4.08/66.69
G	β-d-Gal*p*-(1→	4.50/105.96	3.62/72.24	3.76/72.61	4.19/70.15	3.71/74.46	3.75, 3.92/62.39
H	→6)-β-d-Gal*p*-(1→	4.46/105.03	3.67/71.71	3.72/73.26	4.22/70.20	3.94/75.51	3.92, 4.09/70.24

^a^ Spectra were performed on an Agilent DD2 500M NMR spectrometer. Chemical shifts are referenced to internal acetone at δ_H_ 2.225 and δ_C_ 31.07. Gal*p*: galactopyranose.

**Table 4:** Effect of SGC on insulin resistance.

Groups ^a^	Fasting Insulin Content (mlU/L)	HOMA-IR	HOMA-β	QUICKI
NC	20.31 ± 2.82	4.67 ± 0.40	245.03 ± 73.96	0.495 ± 0.009
MC	57.79 ± 2.81 ^##^	50.38 ± 3.56 ^##^	71.78 ±0.34 ^##^	0.327 ± 0.004 ^##^
PC	30.73 ± 2.79 **	13.28 ± 1.97 **	99.13 ± 0.06 **	0.404 ± 0.011 **
SGC-H	35.80 ± 1.85 ^#^**	16.62 ± 0.30 **	103.30 ± 10.57 **	0.389 ± 0.001 **
SGC-M	46.99 ± 3.13 ^##^	27.14 ± 1.51 ^##^	98.99 ± 8.05 ^#^	0.359 ± 0.003 ^##^
SGC-L	50.56 ± 4.02 ^##^	31.73 ± 3.79 ^##^	95.33 ± 2.49 ^##^	0.351 ± 0.006 ^##^

^a^ NC: the normal control group received standard chow; MC: the diabetic model control group; PC: the positive control group treated with metformin at the dose of 200 mg/kg/day; SGC-L: the low-dose SGC group treated with SGC at the dose of 100 mg/kg/day; SGC-M: the middle-dose SGC group treated with SGC at the dose of 200 mg/kg/day; SGC-H: the high-dose SGC group treated with SGC at the dose of 400 mg/kg/day. Data were expressed as the mean ± SD (*n* = 8). Analysis of fasting insulin content was performed by one-way ANOVA followed by Tukey’s post hoc test. Analyses of HOMA-IR, QUICKI and HOMA-β were performed by non-parametric Kruskal–Wallis test with subsequent Dunn’s post hoc test. ^#^ *p* < 0.05 and ^##^ *p* < 0.01 versus NC group, ** *p* < 0.01 versus MC group.

**Table 5:** Effect of SGC on oxidative stress in the T2DM mice.

Groups ^a^	SOD (U/mL)	CAT (U/mL)	MDA (nM)	GSH (μM)
NC	138.37 ± 6.86	12.55 ± 1.87	9.22 ± 1.25	163.46 ± 7.07
MC	50.54 ± 3.62 ^##^	5.43 ± 0.53 ^#^	16.47 ± 1.63 ^##^	67.19 ± 8.49 ^##^
PC	119.86 ± 6.17 **	10.63 ± 1.86 *	9.79 ± 0.30 **	137.05 ± 4.70 ^#^**
SGC-H	111.99 ± 2.39 ^#^**	10.56 ± 0.64 *	11.47 ± 0.79 *	114.87 ± 3.76 ^##^**
SGC-M	89.46 ± 3.66 ^##^**	8.29 ± 1.31	14.38 ± 1.13 ^#^	84.39 ± 0.83 ^##^
SGC-L	68.96 ± 6.77 ^##^	6.59 ± 0.85 ^#^	14.19 ± 0.97 ^#^	70.80± 6.72 ^##^

^a^ NC: the normal control group received standard chow; MC: the diabetic model control group; PC: the positive control group treated with metformin at the dose of 200 mg/kg/day; SGC-L: the low-dose SGC group treated with SGC at the dose of 100 mg/kg/day; SGC-M: the middle-dose SGC group treated with SGC at the dose of 200 mg/kg/day; SGC-H: the high-dose SGC group treated with SGC at the dose of 400 mg/kg/day. Data were expressed as the mean ± SD (*n* = 8). Inter-group comparisons utilized one-way ANOVA followed by Tukey’s post hoc test. ^#^ *p* < 0.05 and ^##^ *p* < 0.01 versus NC group, * *p* < 0.05 and ** *p* < 0.01 versus MC group.

## Data Availability

Data presented in this study are available on request.

## Supplementary Materials

The following supporting information can be downloaded at: https://www.mdpi.com/article/10.3390/md23100374/s1, Figure S1. NMR spectra of SGC; Figure S2. NMR spectra of dsSGC.
