# Supplementary material for: A Sulfated Polysaccharide from the Green Alga Caulerpa taxifolia: Characteristics of Its Structure and Anti-Diabetic Activity
Source: Mar Drugs. 2025 Sep 25;23(10):374. doi: 10.3390/md23100374 (PMC12565132; doi:10.3390/md23100374)
Supplement: Supplementary file 1 [file marinedrugs-23-00374-s001.zip › marinedrugs-3884165-supplementary.pdf]

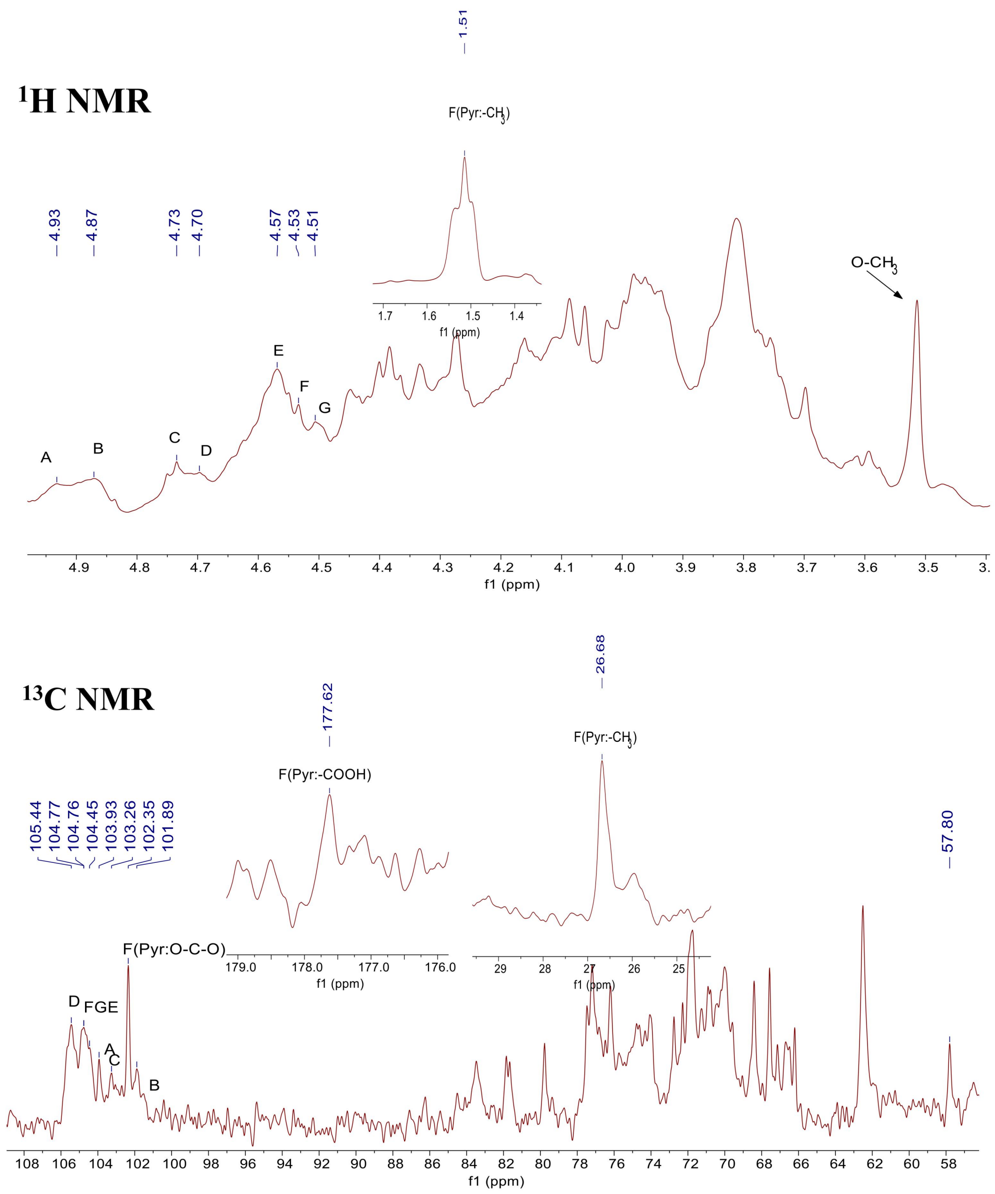

Figure S1. *Cont.*

^{1}H-^{1}H COSY

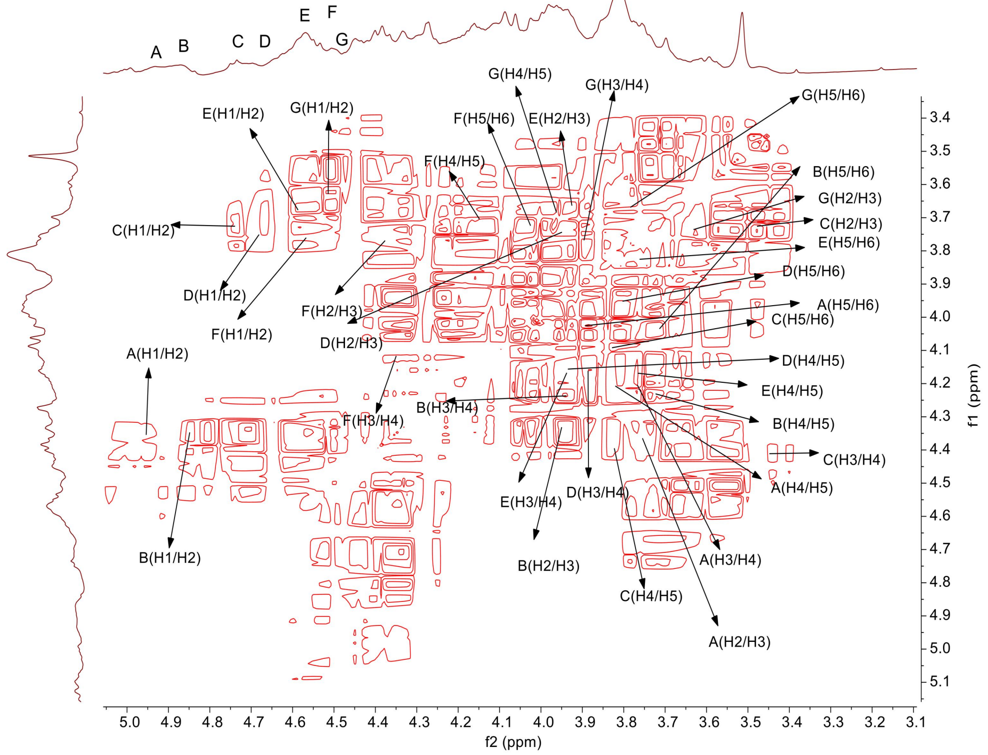

^{1}H-^{13}C HSQC

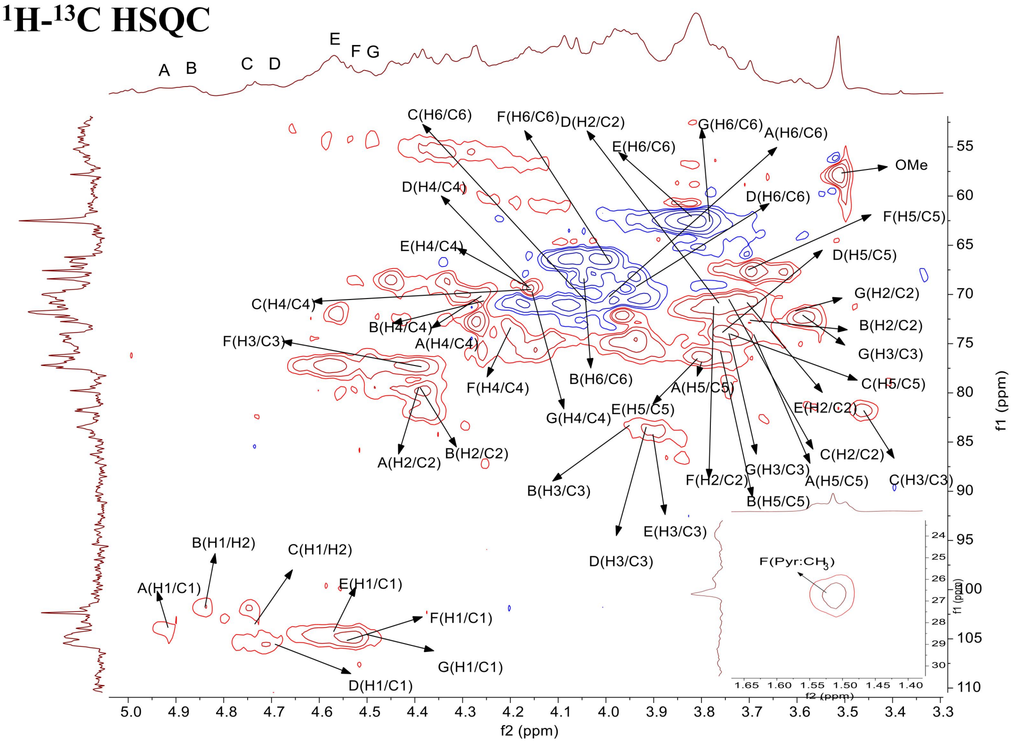

Figure S1. *Cont.*

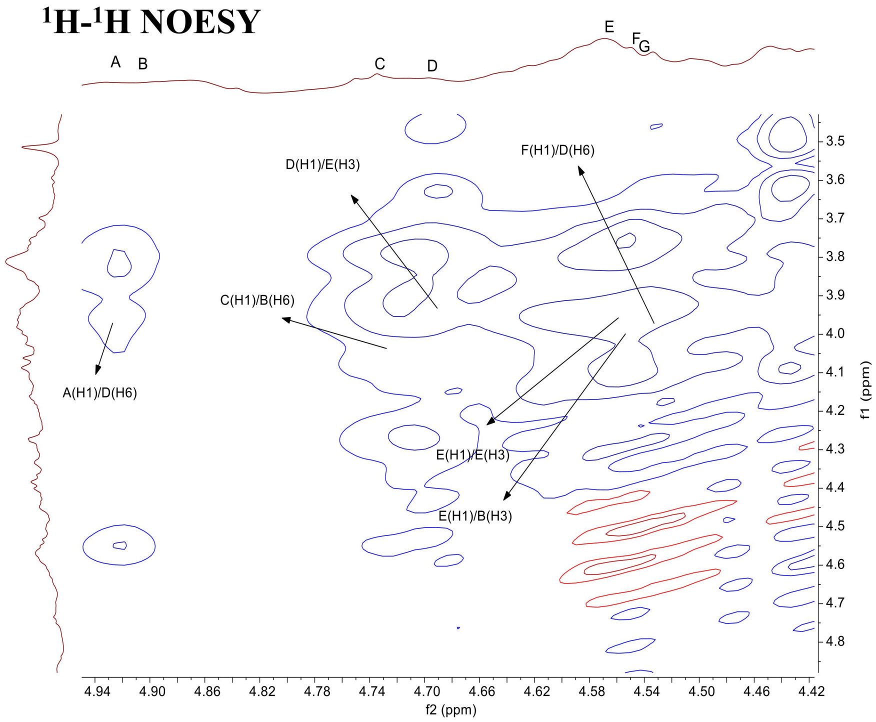

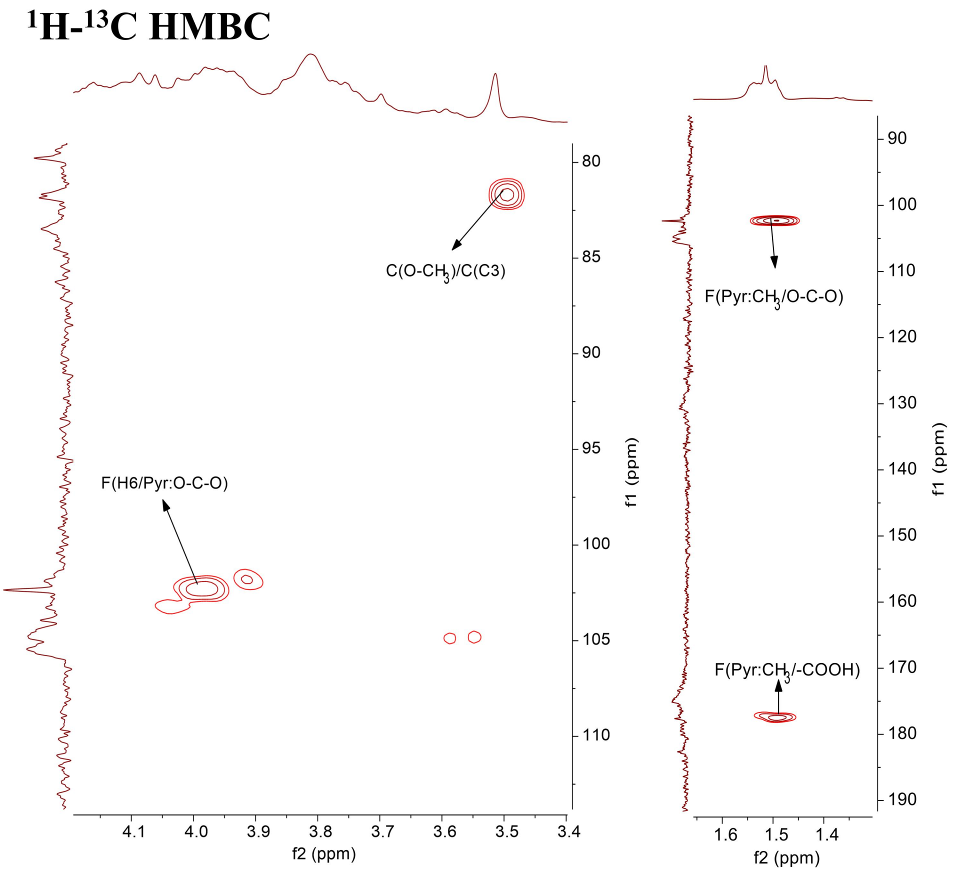

Figure S1. NMR spectra of SGC. A: →6)-β-D-Gal*p*(2SO$_4$)-(1→, B: →3,6)-β-D-Gal*p*(2SO$_4$)-(1→, C: →6)-β-D-Galp(3OMe)-(1→, D: →3,6)-β-D-Gal*p*-(1→, E: →3)-β-D-Gal*p*-(1→, F: →3)-β-D-Gal*p*(4,6-Pyr)-(1→, G: β-D-Gal*p*-(1→. Gal*p*: galactopyranose. Spectra were performed on an Agilent DD2 500M NMR spectrometer. Chemical shifts are referenced to internal acetone at δ_H 2.225 and δ_C 31.07.

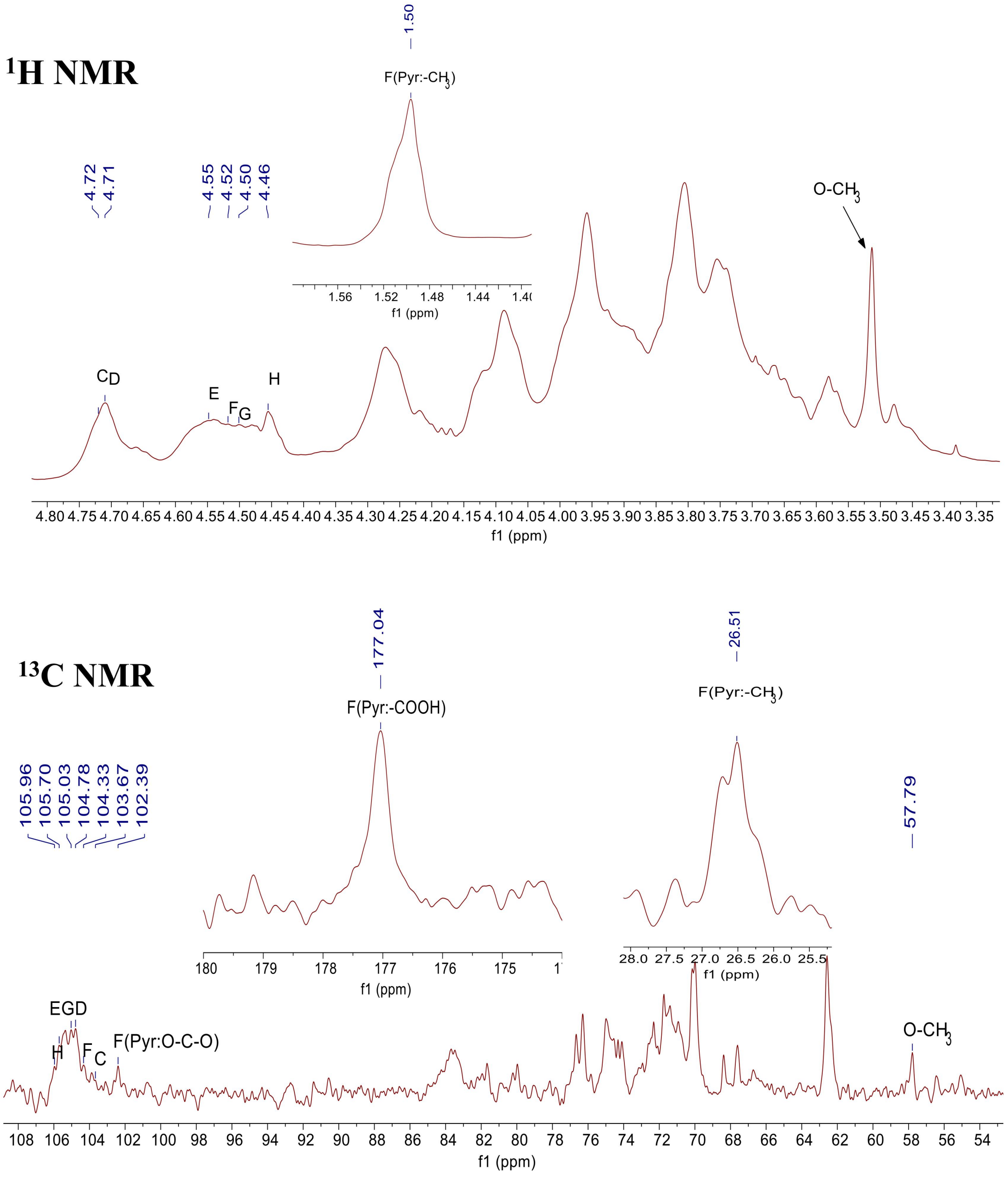

Figure S2. *Cont.*

^{1}H-^{1}H COSY

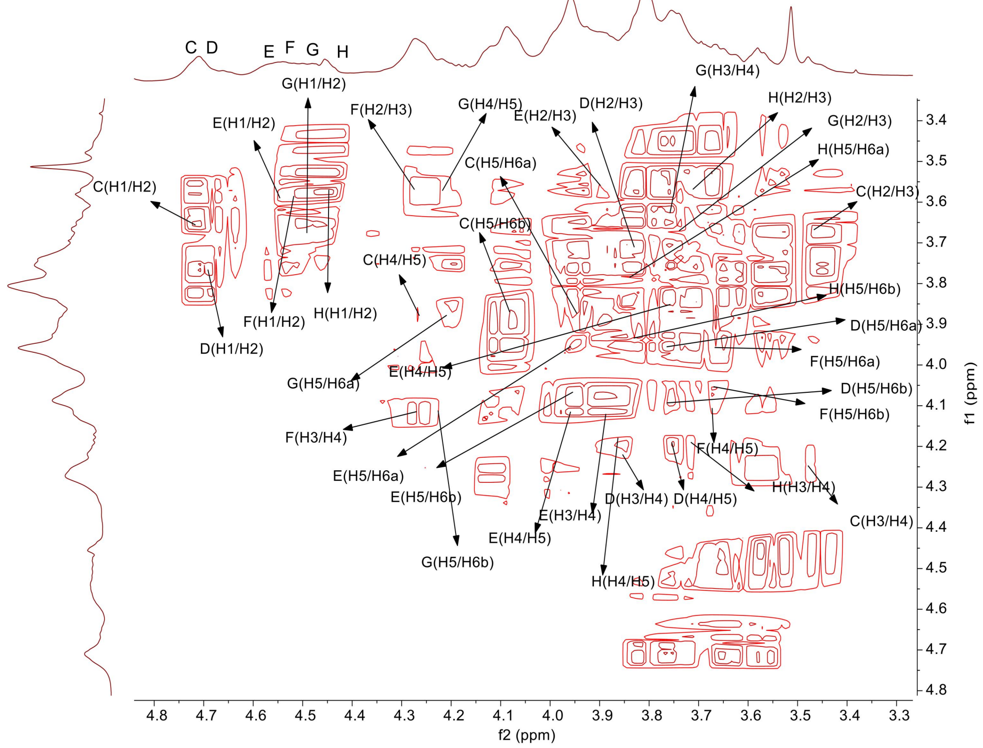

^{1}H-^{13}C HSQC

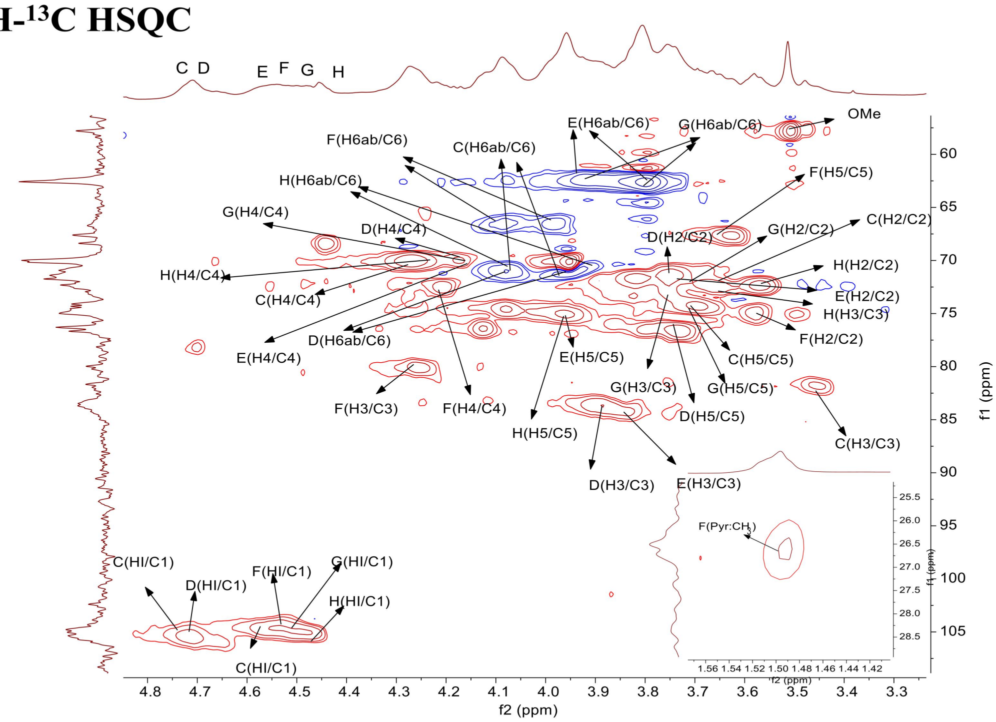

Figure S2. *Cont.*

^1H-^1H NOESY

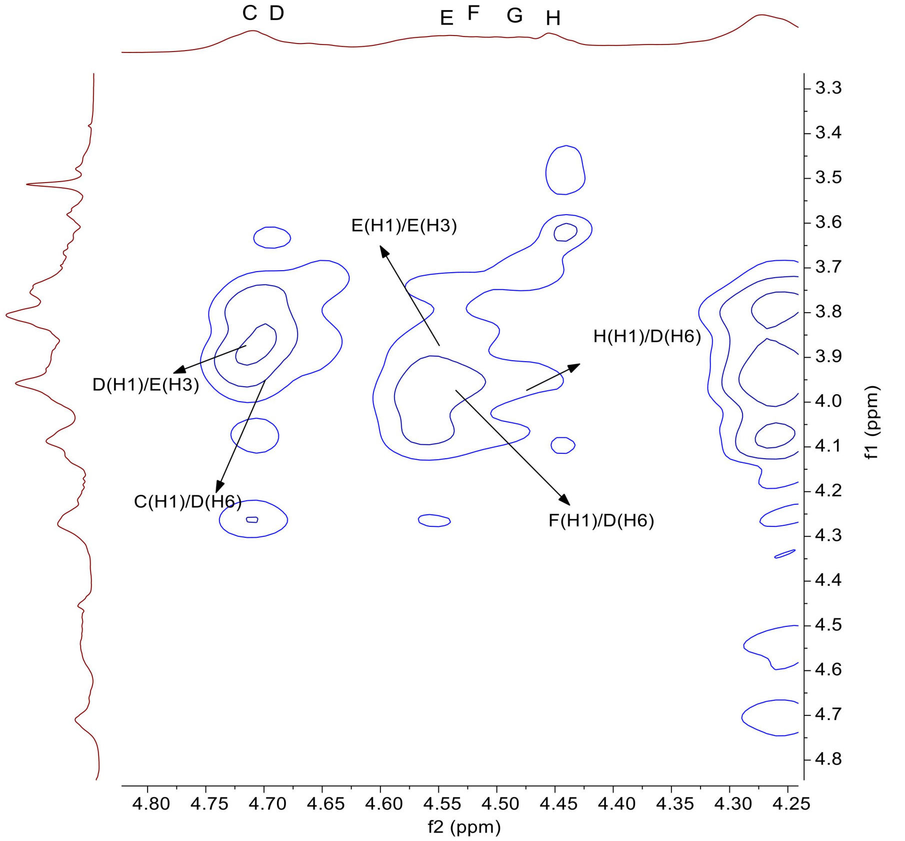

^1H-^{13}C HMBC

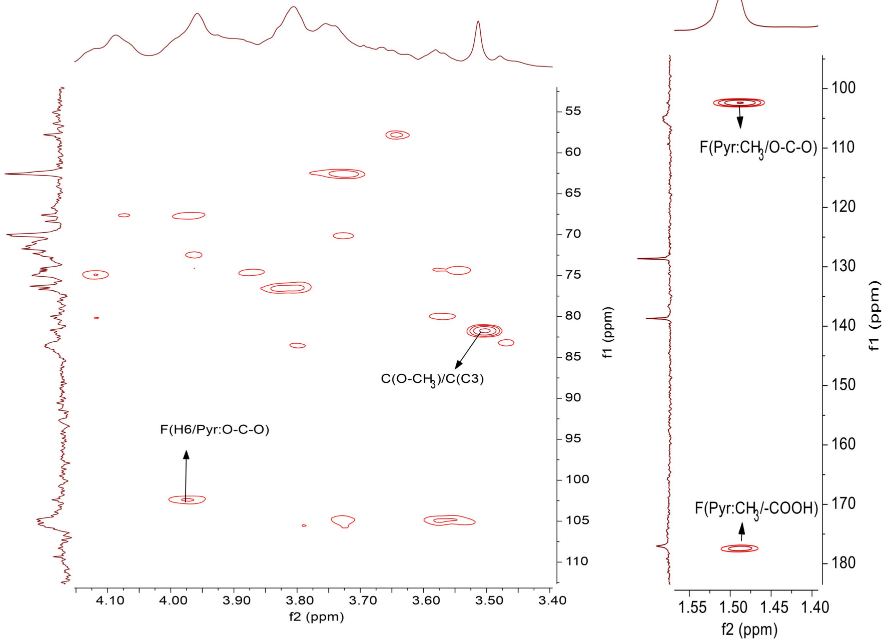

Figure S2. NMR spectra of dsSGC. Spectra were performed on an Agilent DD2 500 MHz NMR spectrometer using acetone as internal standard. C: →6)-β-D-Gal*p*(3OMe)-(1→; D: →3,6)-β-D-Gal*p*-(1→; E: →3)-β-D-Gal*p*-(1→; F: →3)-β-D-Gal*p*(4,6-Pyr)-(1→; G: β-D-Gal*p*-(1→; H: →6)-β-D-Gal*p*-(1→. Spectra were performed on an Agilent DD2 500M NMR spectrometer. Chemical shifts are referenced to internal acetone at δ_H 2.225 and δ_C 31.07.
